# Fatal Meningitis in a 14-Month-Old with Currarino Triad

**DOI:** 10.1155/2016/1346895

**Published:** 2016-08-11

**Authors:** Hanan Mohammed Al Qahtani, Khalid Suliman Aljoqiman, Hisham Arabi, Hesham Al Shaalan, Sumit Singh

**Affiliations:** ^1^Diagnostic and Interventional Neuroradiology, National Guard Health Affairs, Riyadh 11426, Saudi Arabia; ^2^Medical Imaging, National Guard Health Affairs, Riyadh 11426, Saudi Arabia; ^3^General Pediatrics, King Abdullah Specialist Children's Hospital, National Guard Health Affairs, Riyadh 11426, Saudi Arabia; ^4^Pediatric Radiology, King Abdullah Specialist Children's Hospital, National Guard Health Affairs, Riyadh 11426, Saudi Arabia

## Abstract

We report a case of a 14-month-old girl with undiagnosed Currarino triad presenting acutely with meningitis caused by enteric commensals. Head CT demonstrated a large pneumocephalus. A fistulous neurenteric tract through a presacral mass was present on spine MRI and abdominal CT. The patient had a history of constipation for the last three months. However, an underlying diagnosis of Currarino triad had not been suspected. In retrospect, a sickle-shaped sacral anomaly was present on a previous abdominal radiograph. The patient succumbed to complications of meningitis. The purpose of the case report is to highlight the potentially fatal complication of Currarino triad and sensitize radiologists to look actively for sacral anomalies on abdominal radiographs, especially of children with chronic constipation.

## 1. Introduction

Currarino syndrome is a rare congenital complex disorder characterized by triad of partial sacral agenesis, anorectal malformation, and presacral mass [1]. Currarino syndrome has an autosomal dominant inheritance with highly variable expression ranging from asymptomatic to patients with the complete triad [2, 3]. The mutations identified in most familial and some sporadic cases are in the homeobox gene, HLXB9, now called MNX1 gene at chromosome 7q36 [4, 5].

Scimitar or sickle-shaped sacrum is the hallmark of the triad. The sacral abnormality is most often identified on an abdominal or pelvic radiograph. In older children, these radiographs may have been obtained for workup of chronic constipation or urogenital problems which are the most common symptoms of this syndrome [6–8].

It is important to identify this syndrome early to prevent future complications like meningitis, which occurs in 7–11% of cases and has a high mortality of 50% [6, 7].

## 2. Case History

A 14-month-old girl presented to the emergency department (ED) with high fever, abdominal distension, vomiting, and neck stiffness. The patient had a history of chronic constipation which had got worse in the preceding three months. She had delayed gross motor milestones. The immunizations were up-to-date. Her parents were consanguineous. She had four siblings. Her oldest brother had history of surgically repaired anal atresia at birth.

On examination, the patient had clear signs of meningeal irritation. Her abdomen was significantly distended with visible peristalsis.

At presentation she had peripheral leukocytosis (WBC: 26.26 × 10^9^/l, neutrophils 53%, and bands 19%) and thrombocytosis (616 × 10^9^/l). ESR was elevated at 89 mm/hr. Lumbar puncture showed purulent material. The CSF culture results were available on the next day and showed growth of polymicrobes, including extended-spectrum beta-lactamases* E. coli*, in addition to* Enterococcus faecium*. Urine and blood cultures were negative.

**Figure 1:**
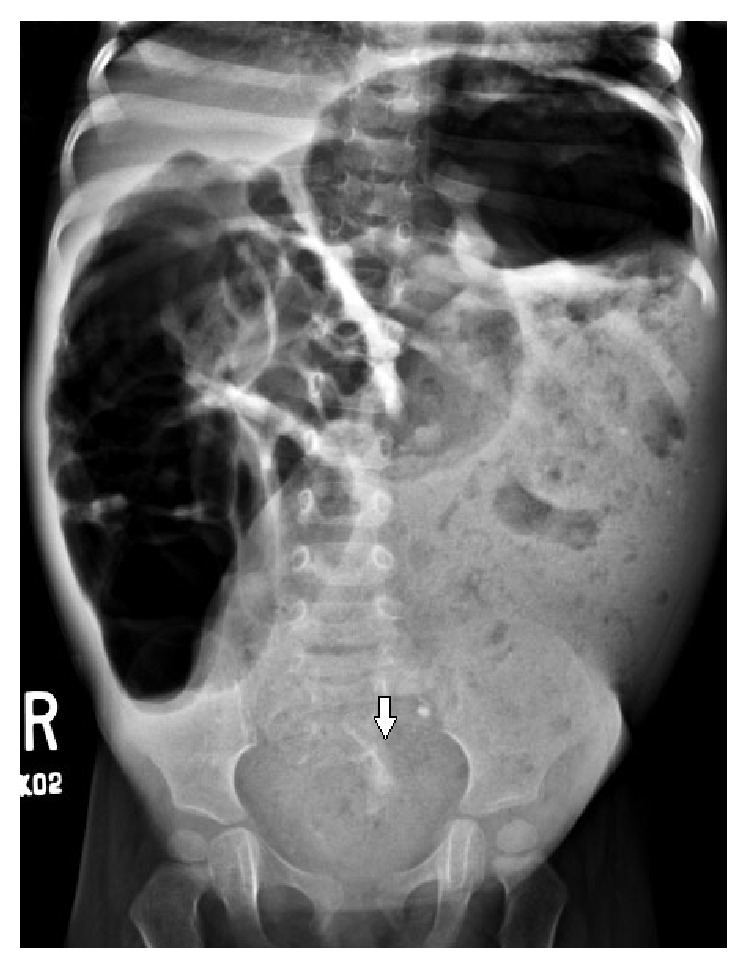
Abdominal radiograph for abdominal distention evaluation. It shows significantly dilated air and fecal filled bowel loops with fecaliths. There is a sickle-shaped sacral defect (arrow), not reported initially.

**Figure 2:**
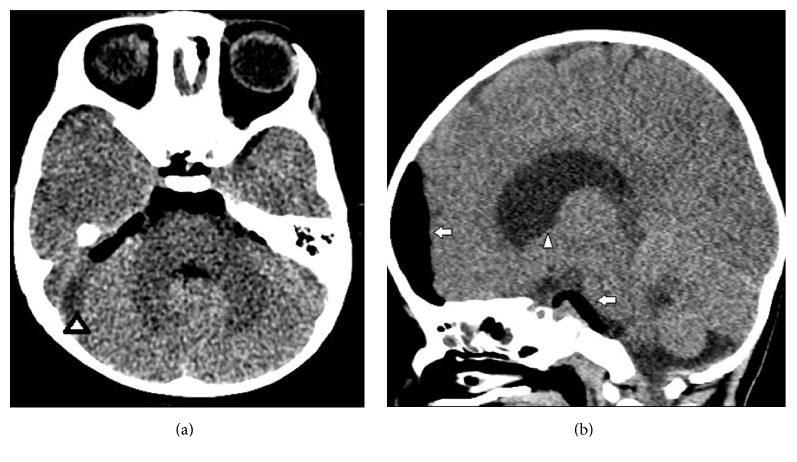
Head CT on day 2 for evaluation of meningitis. (a) Axial head CT shows subdural effusion over the right cerebellar hemisphere abutting the sigmoid sinus (arrowhead) and large air in the prepontine cistern. (b) Sagittal reformat demonstrates large pneumocephalus over the left frontal lobe and in the prepontine cistern (arrows). There is mild lateral ventricle dilatation (arrowhead).

**Figure 3:**
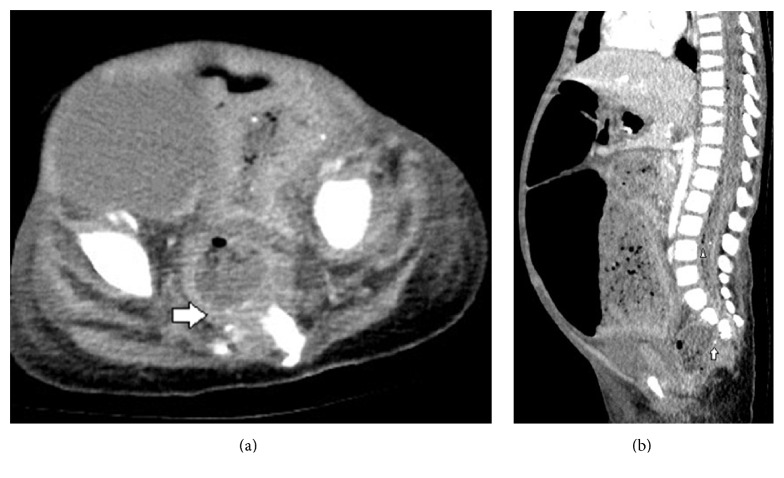
Abdominal CT on day 4 for evaluation of progressive abdominal distention. (a) Axial CT slice through the lower pelvis shows hypoplasia of the sacrum and an indeterminate presacral mass extending into the spinal canal (arrow). The mass contains heterogeneous material and air and the contents have a similar appearance to the fecal material in the bowel. (b) Sagittal abdominal CT reformat again shows the presacral mass in continuity with the sacral canal having a few fecaliths. Notice few fecaliths (arrow) and air (arrowhead) in the spinal canal, suggesting communication with the anal canal.

**Figure 4:**
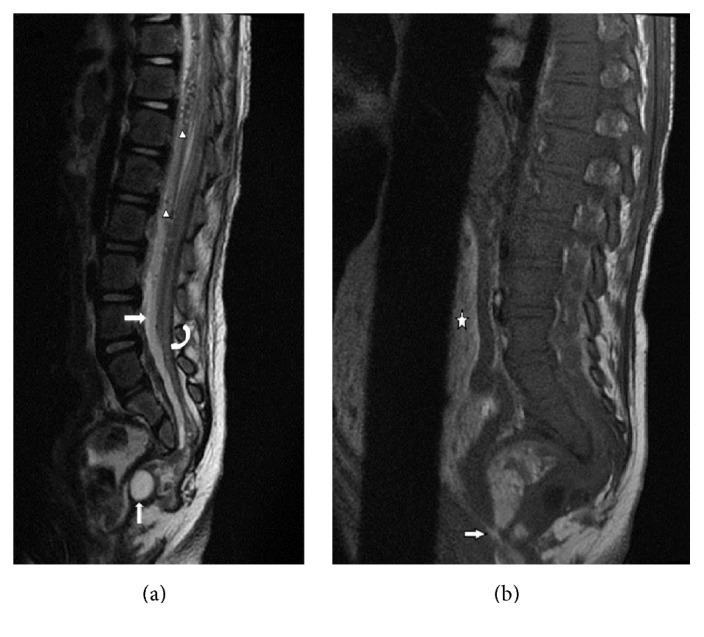
Spine MRI on day 5 to further characterize the presacral mass. (a) Sagittal T2 weighted MR image of the lower spine shows complex presacral mass with a small separate hyperintense cyst communicating with the thecal sac, which could be meningocele (thin arrow). Spinal canal shows epidural fluid collection (arrow) with multiple air foci (arrowheads). There is low-lying conus and a tethered cord, reported in 70% of Currarino syndrome cases (curved arrow). (b) Sagittal T1 weighted MR image of the spine shows the communication of the larger presacral mass with the anal canal (arrow). The contents of the mass have similar intensity to the contents of the dilated sigmoid colon (asterix).

**Figure 5:**
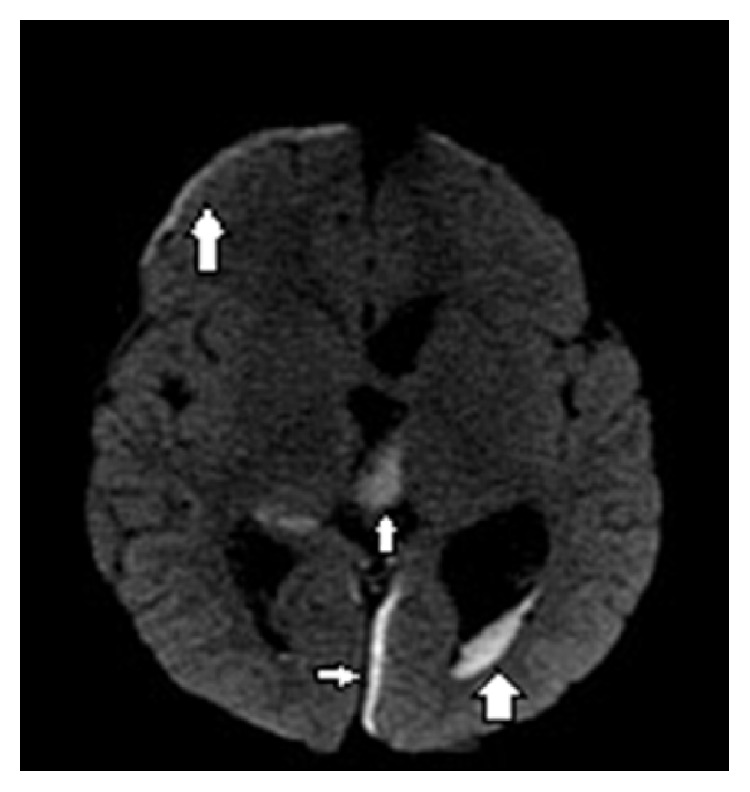
Brain MRI on day 5 for meningitis evaluation. Axial diffusion weighted MR image of the brain shows restricted diffusion over the right frontal lobe and left medial occipital lobe and in the ventricles suggesting subdural empyemas and intraventricular abscesses (arrows).

**Figure 6:**
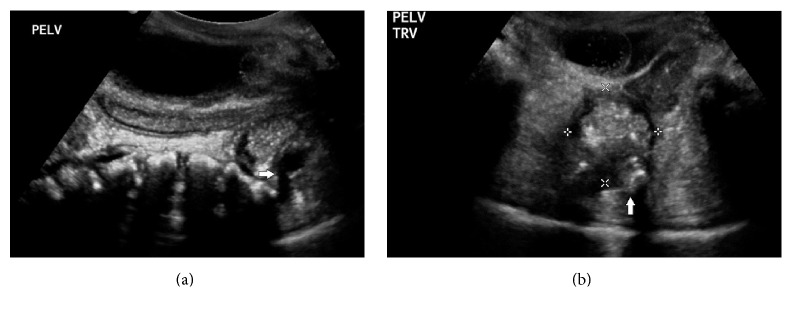
Abdominal ultrasound on day 7 to evaluate the neuroenteric fistula. (a) Gray scale sagittal image demonstrates a complex presacral mass with heterogeneous echogenicity and multiple echogenic foci representing air. There is a hypoechoic cystic structure within it which is in continuity with the sacral canal (arrow). (b) Subsequent transverse gray scale US image shows echogenic foci representing air in the small inferior hypoechoic cyst with the presacral mass (within calipers) suggesting continuous fistulous connection (arrow).

An abdominal radiograph was interpreted as stool filled significantly dilated descending and sigmoid colon (Figure 1). Barium enema was advised to rule out Hirschsprung's disease. CT head showed nontraumatic large pneumocephalus, too disproportionate to be explained by lumbar puncture (Figure 2). Abdominal CT was obtained to investigate progressive abdominal distention and look for neurenteric fistula. It demonstrated a presacral mass, sickle-shaped sacral defect, coccyx agenesis, and a fistulous tract between the sacral canal and the anal canal (Figure 3). The presacral mass communicated with the spinal canal through the sacral defect. The contents of the mass had similar appearance to the fecal material in distended bowel loops and some fecaliths were also seen in the spinal canal suggesting fistulous connection (Figure 3). In retrospect, the sacral defect was seen on the abdominal radiograph (Figure 1). A subsequent spinal MRI revealed a low-lying conus medullaris. The cauda equina nerve roots were bunched together by an epidural collection. The distal thecal sac was in communication with the heterogenous presacral mass containing a hyperintense cyst (Figure 4). The mass and bowel contents had similar signal confirming fistulous communication. Multifocal tiny artifacts from air were seen in the thecal sac and epidural collection suggestive of the connection of the mass with the spinal canal and explaining the source of large pneumocephalus and central nervous system infection (Figure 4). MRI brain demonstrated cerebritis, meningitis, subdural empyema, and intraventricular abscesses (Figure 5). Pelvic ultrasound to evaluate the fistula reaffirmed the neurenteric connection through the presacral mass (Figure 6).

In spite of initiation of broad-spectrum antibiotics and supportive treatment, the patient succumbed to the complications of meningitis and died eight days after admission.

## 3. Discussion

The true incidence of Currarino syndrome is difficult to determine due to high proportion of clinically asymptomatic patients and variability of phenotypic expression. The components of the triad, sacral defect, presacral mass, and anorectal malformation occur with different frequencies although the sacral anomaly is the most common occurring defect [9, 10]. Anal stenosis is the most frequent anorectal malformation followed by imperforate anus [8, 10]. Infundibular anus is characteristically described with the syndrome, although it was not seen in this patient. Chronic intestinal pseudoobstruction is part of the CS, but no precise defect either in intrinsic innervation or in muscular coats of the hindgut has been discovered [8]. Occult spinal dysraphism and spinal cord anomalies including syrinx, low-lying conus, diastematomyelia, spinal lipoma, and tethered cord are frequent [10].

The presacral mass can be anterior meningocele, teratoma, dermoid, or epidermoid cysts [7, 10]. The presacral mass is the cause of most symptoms and complications of the syndrome. The local mass effect not only contributes to constipation but also can lead to urogenital symptoms, local infection, and pain. The mass could be a teratoma which can occasionally turn malignant [8, 10]. The presacral mass can erode into the rectum leading to neuroenteric fistulous communication resulting in fatal meningitis and large pneumocephalus. However, the origin of neuroenteric fistula is most often congenital as compared to tumoral evolution (malignant degeneration or chronic mass effect). In fact, the presence of atypical organisms (enteric commensals in our case) in an immunocompetent child should prompt a search for anatomic defects as the source of the infection [11]. Moreover, in a child with atypical meningitis and large unexplained nontraumatic pneumocephalus, neurenteric fistula as a possible etiology needs to be strongly considered.

Currarino syndrome is caused by abnormality in the canalization and retrogressive differentiation of caudal cell mass formed by secondary neurulation [12, 13]. The lower vertebral column formation is also less well organized and forms from a mass of cells composed of notochord, mesenchyme, and neural tissue which divide to form sacral and coccygeal somites. The cloaca, which forms the lower genitourinary and anorectal tracts, lies in close proximity ventral to the caudal cell mass separated from it by notochord. As a result anorectal and urogenital malformations are often associated with vertebral abnormalities like lumbosacral agenesis/hypogenesis or spinal cord anomalies like terminal myelocystocele and tethered spinal cord [12, 13].

Notochord is important in the induction of thoracic and abdominal viscera as well as the neural tube. Therefore, abnormal notochord formation may lead to wide range of anomalies involving upper GI tract, respiratory tract, abdominal viscera, vertebra, and spinal cord as seen in VACTERL association, OEIS (omphalocele, exstrophy, imperforate anus, and spinal deformities), and the Klippel-Feil syndrome [14]. Caudal regression syndrome is believed to result from disturbances of caudal mesoderm, the caudal cell mass, and cloaca prior to 4-week gestation. The agenesis of the caudal spinal cord and spinal column is thought to be due to toxins, maternal diabetes, ischemia, infection, or genetic predisposition leading to disruption of normal mesoderm development and migration [15].

As indicated by embryology, a high correlation of lumbosacral defect with anorectal and urogenital malformations is well described [10, 16, 17]. This further emphasizes the value of lower spine column and spinal cord scrutiny in children with genitourinary and gastrointestinal systems.

The identification of sacral anomaly in an older child with constipation is challenging as the defect can be obscured by large fecal load in distal colon and rectum. A diligent evaluation of sacral spine on abdominal radiograph, especially in children with chronic lower gastrointestinal and urogenital symptoms, can lead to better detection or a dedicated sacral radiograph can be obtained. The sacral defect is sometimes identified on images obtained for vesicocystourethrogram. It is imperative to get a spine or pelvic MRI when a bony sacral defect is detected. The presacral mass when present is ideally removed surgically to prevent future complications.

Patients with Currarino triad who are asymptomatic or have less severe chronic complaints and no visible anorectal malformation pose a clinical and radiological diagnostic challenge. The disease is rare enough to not warrant early consideration. These patients may present late with unexpected serious complications like meningitis or malignant degeneration of presacral mass. The first-degree relatives of patients with Currarino triad should be offered pelvic radiograph to identify asymptomatic heterozygotes of this autosomal dominant, variable penetrance disorder. Once a mutation has been detected, all family members should undergo genetic counseling to raise awareness and for pregnancy planning [4]. The oldest brother of our patient had a history of surgically treated anorectal malformation which may have been an expression of this syndrome. The family has been offered genetic tests and screening by pelvic radiographs.

Our patient deteriorated rapidly and became unfit for surgery. In literature, appropriate management of these patients has been described by a two-step operation. The first step is to perform a functional colostomy to cut out the source of infection. The second stage involves definitive repair of the fistula and anorectal malformation along with tumor resection by posterior sagittal anorectoplasty after three months of antibiotic therapy [3, 5, 10].

## 4. Conclusion

We emphasize the importance of careful evaluation of lower spine on abdominal radiographs of children with chronic constipation or urogenital complaints. Dedicated sacral radiographs can be done, if lower spine is not visualized optimally on abdominopelvic radiographs. Apart from detecting other disorders, it may lead to early identification of Currarino triad, thus preventing possible future serious complications. It is important to evoke the diagnosis of Currarino triad in the setting of meningitis with abnormal sacrum. These cases should be evaluated emergently by MRI and lumbar puncture with wariness. Early appropriate treatment can reduce morbidity and mortality.
